# Disparities in out-of-pocket costs for disease-modifying therapy under Japan's universal health insurance system

**DOI:** 10.1016/j.tjpad.2025.100170

**Published:** 2025-04-15

**Authors:** Kenichiro Sato, Ryoko Ihara, Yoshiki Niimi, Saki Nakashima, Atsushi Iwata, Takeshi Iwatsubo

**Affiliations:** a Department of Neuropathology, Graduate School of Medicine, The University of Tokyo, Tokyo, Japan; b Unit for Early and Exploratory Development, The University of Tokyo Hospital, Tokyo, Japan; c Dementia Inclusion and Therapeutics, The University of Tokyo Hospital, Tokyo, Japan; d Department of Neurology, Tokyo Metropolitan Institute of Geriatrics and Gerontology, Tokyo, Japan; e Department of Healthcare Economics and Health Policy, Graduate School of Medicine, The University of Tokyo, Tokyo, Japan; f Department of Neurology, Graduate School of Medicine, The University of Tokyo, Tokyo, Japan

Dear editor,

The approval of anti-amyloid monoclonal antibodies (mAbs), including lecanemab and donanemab, offers new therapeutic options for patients with Alzheimer's disease (AD), targeting AD pathology to slow disease progression. However, their high cost raises concerns about the sustainability of healthcare systems [1], as well as the possibility of substantial financial burden for individual patients receiving mAb therapies.

Japan's universal health insurance system mandates enrollment for all residents and provides coverage for a wide array of treatments and services. One key feature is the High-Cost Medical Care Benefit (HCMCB) program [2], which caps monthly out-of-pocket medical expenses to prevent financial hardship for patients — particularly for older adults receiving pensions. This program is essential for patients receiving expensive treatments, such as immune checkpoint inhibitor drugs, molecular targeted drugs for cancer and now mAbs for early-stage AD, by limiting a patient's monthly share of costs once a certain threshold has been reached.

However, we are concerned that the current HCMCB cost-sharing arrangements might lead to treatment disparities among patients with AD. Fig. 1 illustrates the relationship between annual household income (x-axis) and the out-of-pocket expenses (y-axis) for anti-amyloid mAbs infusion and scheduled brain MRI scans within the first 12 months of treatment for 60-kg patients under age 70, who must pay a 30 % co-payment. Under the current HCMCB program, patients with AD will face financial burdens that vary depending on their household income: those in the younger population, who earn pre-tax household incomes of US$22,000–85,000 (purchasing power parity, PPP) in the previous year, will have to pay approximately US$6000–7000 (PPP). Meanwhile, individuals with higher household incomes (> US$85,000 PPP) sometimes do not meet the thresholds for reduced co-payments, resulting in annual out-of-pocket expenses for DMT therapy that can surpass US$10,000 (PPP) in the first year.

**Fig. 1:**
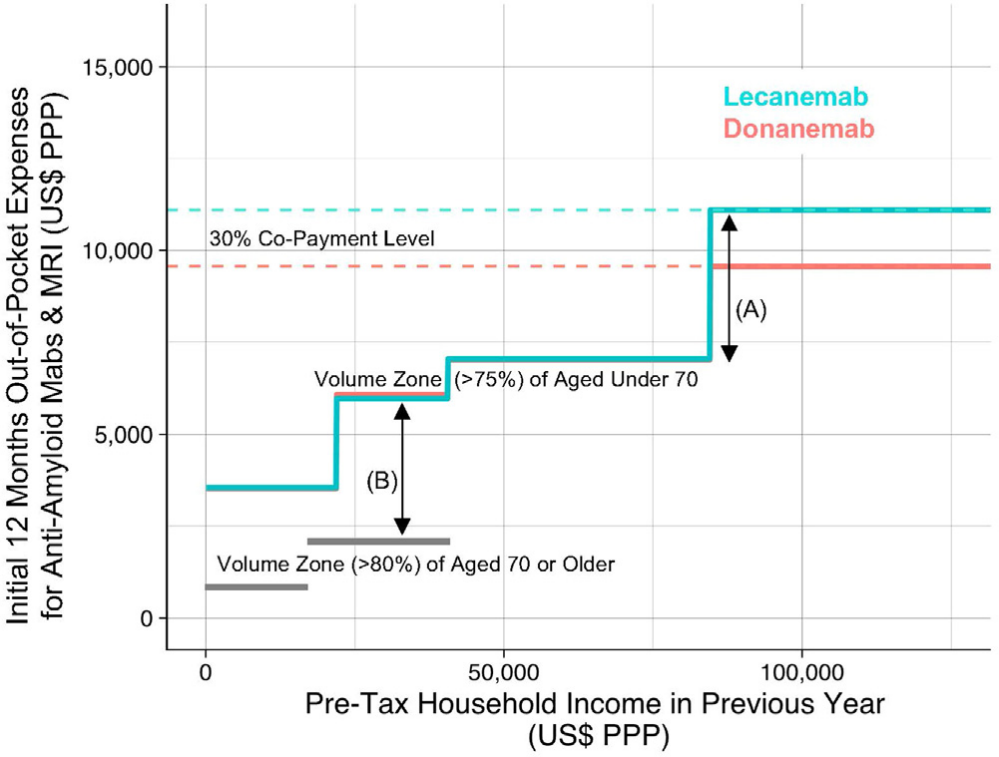
Out-of-pocket expenses (approximate sum) for aged under 70, determined by the household income. The relationship between yearly pre-tax household income (x-axis) and the yearly out-of-pocket expenses (y-axis, approximate sum) under the current HCMCB program [5], for anti-amyloid mAb infusion and scheduled brain MRI scans within the first 12 months of treatment for 60-kg patients aged under 70. These out-of-pocket expenses were calculated by the authors under the following assumptions: 1) patients’ household income remained fixed for the recent two years, 2) body weight is fixed throughout the 12 months of infusion, 3) amyloid status was confirmed by amyloid PET scan (raw cost JP¥146,100 /time) one-month before initiating anti-amyloid mAb infusion, 4) brain MRI scans (raw cost JP¥17,800 /time) were conducted only according to the recommended schedules, 5) any other medical care cost is not considered for calculation, 6) anti-amyloid mAb infusion is conducted at outpatient clinic only, 7) infusion of lecanemab (raw cost JP¥45,777 / 200 mg) and donanemab (raw cost JP¥66,498 / 350 mg) is conducted 24 and 12 times in the first year, respectively, 8) no in-hospital care is required throughout the period, and 9) other factors such as household information, year-end adjustments, and the place of residence are not taken into account. Thus the expense calculation is not entirely accurate. The annual income of households exempt from resident tax was set at less than JP¥2000,000 for visualization purposes. Households receiving public welfare assistance are not considered in this figure. For ease of understanding of readers, JP¥ is converted to US$ at the rate of US$1 (PPP) = JP¥91 in 2024. Abbreviations: BW, body weight; mAb, monoclonal antibody; MRI, magnetic resonance imaging; PPP, purchasing power parity; HCMCB, High Cost Medical Care Benefit; PET, positron emission tomography.

A “step-like” increase in financial responsibility, reflecting the regulations of the HCMCB program, which depends on specific income-based cutoff points, adds another notable characteristic (Fig. 1A). For example, if the household income in the previous year for patients aged under 70 exceeds the threshold of approximately US$85,000 (PPP) even by US$1, they would see their annual medical expenses rise by more than US$2500 (PPP) compared to not exceeding the threshold. Thus, merely crossing an income threshold can trigger a marked jump in out-of-pocket costs. Individuals just above the cutoff point have to bear higher costs than those just below it, possibly leading to a higher likelihood of choosing not to treat despite having comparable clinical needs. Changes in medical costs triggered by steep co-payment thresholds are documented in other disease areas [3].

This income-based burden weighs especially heavily on younger patients—those who are still working or supporting families, while the majority of patients with AD aged 70 or older are subject to different calculations that provide broader cost-sharing assistance (Fig. 1B). Early-onset AD, which affected an estimated 19,000 patients in Japan in 2018 [4], often disrupts employment and family life at a stage when individuals may be managing mortgages or educational expenses for children. As a result, these patients may be more likely to consider forgoing or delaying anti-amyloid mAb treatment, prompted by financial rather than clinical considerations. This phenomenon, described as an aspect of “financial toxicity” [5], highlights the potential financial challenges accompanying costly medical procedures. Such decisions are troubling from a fairness perspective and may compromise the goal of timely, effective care.

Moreover, as Japan's population continues to age and healthcare costs rise, policy adjustments to the HCMCB program were announced in late 2024 to tighten eligibility and raise thresholds within the next two years, although they were eventually frozen in March 2025 for an indefinite period. While such measures could help sustain social security in a broader sense, they may also intensify existing cost-sharing disparities for patients outside the lower-income or older-adult categories. Households classified as “high-income” will receive less financial protection, even though progressive taxation already takes proportionally more from higher-income households. It thus becomes increasingly important to ask whether the “high income” label accurately reflects these families’ ability to manage notable extra costs for advanced therapies.

To address these challenges, reevaluating the existing cost-sharing framework is crucial—especially by offering more targeted support that considers both age and a deeper look at household finances. Shifting from a sliding scale system that adjusts out-of-pocket maximums on a “step-like” graduated basis to a more continuous adjustment mechanism, may help ensure fairer access to mAb therapies.

While our discussion primarily focuses on Japan's universal health insurance system and its specific cost-sharing structure, the challenges we identify—namely, the financial toxicity of high-cost therapies and the potential for income-based disparities in treatment access—are also applicable to other countries with varying models of healthcare financing and insurance coverage, grappling with how to ensure equitable access to expensive treatments.

## Funding

This study was supported by JSPS KAKENHI grant number JP24K10653 (RI, KS), AMED grant number JP23dk0207048 (TI) and JP24dk0207054 (YN). The sponsors had no role in the design and conduct of the study; collection, analysis, and interpretation of data; preparation of the manuscript; or review or approval of the manuscript.

## CRediT authorship contribution statement

**Kenichiro Sato:** Writing – original draft, Visualization, Formal analysis, Conceptualization. **Ryoko Ihara:** Writing – review & editing, Investigation, Conceptualization. **Yoshiki Niimi:** Writing – review & editing, Conceptualization. **Saki Nakashima:** Writing – review & editing, Investigation. **Atsushi Iwata:** Writing – review & editing, Validation. **Takeshi Iwatsubo:** Writing – review & editing, Validation, Supervision.

## Declaration of competing interest

The authors declare the following financial interests/personal relationships which may be considered as potential competing interests:

Ryoko Ihara reports funding from the Japan Society for the Promotion of Science, and a relationship with Eisai and Eli Lilly that includes lecture and advisory fees. Kenichiro Sato reports funding from the Japan Society for the Promotion of Science. Yoshiki Niimi reports funding from the Japan Agency for Medical Research and Development. Atsushi Iwata reports a relationship with Eisai and Eli Lilly that includes lecture and advisory fees. Takeshi Iwatsubo reports funding from the Japan Agency for Medical Research and Development, and a relationship with Eisai and Eli Lilly that includes lecture and advisory fees. If there are other authors, they declare that they have no known competing financial interests or personal relationships that could have appeared to influence the work reported in this paper.

## References

[1] Jönsson L., Wimo A., Handels R., Johansson G., Boada M., Engelborghs S., Frölich L., Jessen F., Kehoe P.G., Kramberger M., de Mendonςa A., Ousset P.J., Scarmeas N., Visser P.J., Waldemar G., Winblad B. (2023). The affordability of lecanemab, an amyloid-targeting therapy for Alzheimer's disease: an EADC-EC viewpoint. Lancet Reg Health Eur.

[2] Reich M.R., Shibuya K. (2015). The future of Japan's health system–sustaining good health with equity at low cost. N Engl J Med.

[3] Fukushima K., Mizuoka S., Yamamoto S., Iizuka T. (2016). Patient cost sharing and medical expenditures for the elderly. J Health Econ.

[4] Awata S., Edahiro A., Arai T., Ikeda M., Ikeuchi T., Kawakatsu S., Konagaya Y., Miyanaga K., Ota H., Suzuki K., Tanimukai S., Utsumi K., Kakuma T. (2020). Prevalence and subtype distribution of early-onset dementia in Japan. Psychogeriatrics.

[5] de Souza J.A., Yap B.J., Hlubocky F.J., Wroblewski K., Ratain M.J., Cella D., Daugherty C.K. (2014). The development of a financial toxicity patient-reported outcome in cancer: the COST measure. Cancer.

